# Population Pharmacokinetics and Exposure–Safety Relationship of Paclitaxel Liposome in Patients With Non-small Cell Lung Cancer

**DOI:** 10.3389/fonc.2020.01731

**Published:** 2021-02-05

**Authors:** Haiyan Zhou, Jiaqing Yan, Wei Chen, Jun Yang, Min Liu, Yuan Zhang, Xin Shen, Yinglin Ma, Xingsheng Hu, Yan Wang, Kehe Du, Guohui Li

**Affiliations:** ^1^Department of Pharmacy, National Cancer Center/National Clinical Research Center for Cancer/Cancer Hospital, Chinese Academy of Medical Sciences and Peking Union Medical College, Beijing, China; ^2^Department of Medical Oncology, National Cancer Center/National Clinical Research Center for Cancer/Cancer Hospital, Chinese Academy of Medical Sciences and Peking Union Medical College, Beijing, China; ^3^Quality Assurance Department, Iphase Pharma Services, Beijing, China

**Keywords:** paclitaxel liposome, population pharmacokinetics, exposure–safety relationship, model, non-small cell lung cancer

## Abstract

**Purpose:**

Paclitaxel liposome (Lipusu) is the first commercialized liposomal formulation of paclitaxel. There has been little data collected on the pharmacokinetics (PK) of paclitaxel liposome, especially in relation to patient use. This study aimed to build a population pharmacokinetic (PopPK) model and further explore the exposure–safety relationship for paclitaxel liposome in patients with non-small cell lung cancer (NSCLC).

**Methods:**

Data from 45 patients with a total of 349 plasma concentrations were analyzed. The PopPK model was built using the non-linear mixed effect modeling technique.

**Results:**

The PK of paclitaxel liposome were well described by a three-compartment model with first-order elimination. For a dose of 175 mg m^–2^, the estimated clearance of total plasma paclitaxel was 21.55 L h^–1^. Age, sex, body weight, total bilirubin, albumin, serum creatinine, and creatinine clearance did not influence the paclitaxel PK. Exposure to paclitaxel had no significant change in the presence of the traditional Chinese medicine, aidi injection. The exploratory exposure–safety relationship was well described by a generalized linear regression model. Higher probabilities of grade >1 neutropenia were observed in patients with higher exposure to paclitaxel.

**Conclusion:**

This PopPK model adequately described the PK of paclitaxel liposome in patients with NSCLC. Predicted exposure of paclitaxel did not change in the presence of the traditional Chinese medicine, aidi injection. The exposure–safety analysis suggested that a higher risk of neutropenia was correlated with higher exposure to paclitaxel.

## Introduction

Liposome, like other nanoparticles (1–3), had been widely developed to deliver various drugs due to its good permeability and stability. It can also improve the solubility of drugs, prolong the residence time in circulation, and increase accumulation in tissues. Paclitaxel liposome (Lipusu^®^) is the first commercialized liposomal formulation of paclitaxel, which was developed by Luye Sike Pharmaceutical (Nanjing, Jiangsu, China). In this formulation, paclitaxel was encapsulated by liposomes, 400 nm in diameter, composed of lecithin and cholesterol (4). It overcame the issues of paclitaxel insolubility and replaced Cremophor EL, which could cause severe hypersensitivity reactions (5). This preparation was approved by the State Food and Drug Administration of China in 2006. To date, it has been widely used in the treatment of various tumors including breast (6), gastric (7), ovarian, and non-small cell lung cancer (NSCLC) (8, 9).

In combination with cisplatin, Lipusu and Cremophor-based paclitaxel are both standard therapies for lung squamous cell carcinoma. Preclinical data has shown that Lipusu had similar antitumor activity to that of Cremophor-based paclitaxel (10). In clinical studies, researchers compared the efficacy and toxicities between Lipusu and Cremophor-based paclitaxel. These results demonstrated that there were comparable efficacies and lower incidence rates of serious hypersensitive reactions in the Lipusu group, but the incidence of other toxicities associated with paclitaxel, such as neutropenia, neurologic toxicities, and alopecia, had no significant difference (6, 7, 11). These adverse effects can still potentially limit dose intensification and impact drug efficacy. However, a few studies reported the pharmacokinetic (PK) profile of Lipusu in patients and its potential relations to toxicities. In addition, a traditional Chinese medicine, aidi injection, as an adjunctive treatment of paclitaxel-based chemotherapy for NSCLC, is now widely used in clinical practices (12, 13). Co-administration of Aidi injection has raised the potential issue of drug–drug interaction. At present, the effect of aidi injection on Lipusu PK is not fully understood. Therefore, in this study, we collected plasma samples from patients with lung squamous cell carcinoma after intravenous administration of Lipusu. We tried to develop a population pharmacokinetic (PopPK) model to characterize the PK profile of Lipusu, and to further evaluate the effect of various factors, including co-administration with aidi injection, on its PK. The exposure–safety analysis was also investigated.

## Materials and Methods

### Patients

Study data were collected from patients in Cancer Hospital, Chinese Academy of Medical Sciences, from June 2015 to August 2018, who were enrolled in a single-center clinical trial. All participants provided written informed consent. All procedures performed in this study were in accordance with the Good Clinical Practice guidelines and the Declaration of Helsinki. The protocol was approved by the Institutional Review Board of Cancer Hospital, Chinese Academy of Medical Sciences, Beijing, China (approval number: 15-123/1050). This study was registered in the Chinese Clinical Trial Registry (registry number: ChiCTR2000029106).

In our study, all the patients had cytologically or histologically confirmed squamous cell NSCLC. Only one patient had also been reported there exist some localized cells with adenoid-like features. They were required to have Karnofsky Performance scale score greater than 70, have symptoms that were recurrent after surgery, or to have had no systematic therapy before. All the subjects had to be aged 18–70 years with an expected survival period longer than 3 months. Participants also had to meet the following inclusive criteria: they had no radiotherapy or other chemotherapy within 3 weeks prior to and throughout the study; had adequate hematopoietic function with absolute neutrophils counts higher than 1.5 × 10^9^ L^–1^ and platelet counts higher than 100 × 10^9^ L^–1^, and adequate hepatic and renal function with alanine transaminase (ALT), aspartate aminotransferase (AST), and total bilirubin and creatinine less than 2.5-folds of upper limit values; and they should not have taken hepatic metabolism enzyme inducers or inhibitors within 4 weeks of the trial. The subjects who had active infections, serious medical diseases, or were accompanied by other types of tumors were excluded.

### Study Design

The patients received an intravenous infusion of paclitaxel liposome over 3 h at 175 mg m^–2^ on the first day, followed by a dose of cisplatin at 75 mg m^–2^ or carboplatin at 4–5 area under the curve (AUC) on the second day in a 3-week cycle. The actual administration dose of paclitaxel liposome was rounded to the nearest vial size. The premedication prior to paclitaxel liposome contained dexamethasone (10 mg, ivgtt), diphenhydramine (50 mg, im), and cimetidine (300 mg, ivgtt). In the second cycle, aidi injection (compound preparation, 100 mL) was administrated prior to chemotherapy. The rest of the medication was the same as that in the first cycle.

Sparse sampling was applied in this study. Blood samples (4 mL) were taken at 1.5 h (during the infusion), 3 h (immediately at the end of infusion), 4 h, 6 h, and 21 h after the administration of paclitaxel liposome. The total paclitaxel concentrations, including encapsulated and unencapsulated paclitaxel, were measured because the encapsulated part was found to be indistinguishable from the total plasma paclitaxel. Plasma samples were separated by centrifugation at 1,000*g* at 4°C and kept at −80°C until analysis.

### Sample Analysis

**FIGURE 1:**
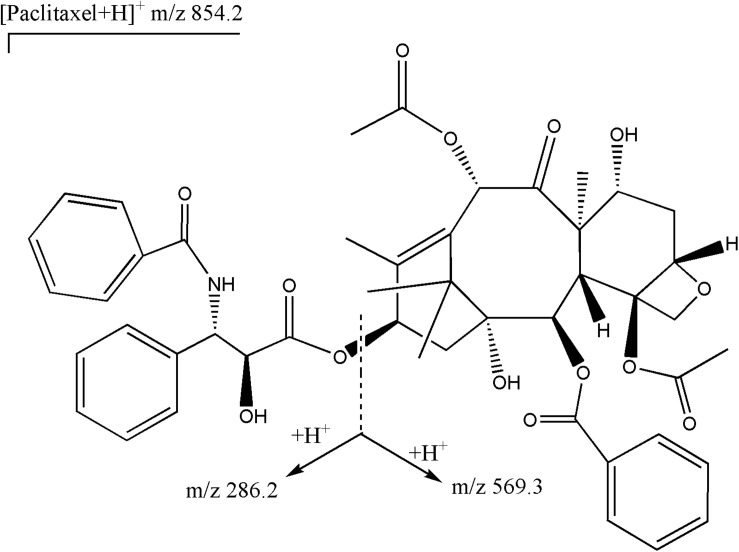
Structures of parent ion (m/z 854.2) and daughter ions (m/z 286.2 and m/z 569.3) for paclitaxel.

A validated liquid chromatography-mass spectrometry/mass spectrometry (LC-MS/MS) method for the determination of total plasma paclitaxel was applied (14). The high-performance liquid chromatographic separation was carried out on a reversed-phase C18 column (3.0 μm, 3.0 mm × 75 mm, Imatakt Unison UK, Japan) with a mobile phase of methanol–water (containing 0.05% formic acid). The total chromatographic run time was 4.5 min. Mass detection was operated on an API 5500 triple-quadrupole mass spectrometer with an electrospray interface (AB MDS Sciex, Applied Biosystems, United States). The settings of mass spectrometry were as follows: spray voltage 3.0 kV; source temperature 500°C; ion source gas1 (GS1) 40 psi, ion source gas2 (GS2) 40 psi; curtain gas (CUR) flow 25 psi; and collision gas (CAD) 7 psi; declustering potential (DP) 60 eV; enterance potential (EP) 10eV; collision energy (CE) 25 eV and collision cell exit potential (CXP) 17 eV. Nitrogen was used as the nebulizer and collision gas. Quantification was performed with multiple reaction monitoring (MRM) mode based on the transitions of m/z 854.2→286.2 for paclitaxel. The qualitative analysis was performed based on the transition of m/z 854.2→569.3. The structures of parent and daughter ions had been shown in Figure 1. Data were collected and analyzed by AB Sciex Analyst software Version 1.6.2. The assay was linear over a concentration range of 10–10,000 ng mL^–1^ with a lower limit of quantification of 10 ng mL^–1^. The extraction recoveries of paclitaxel were in the range of 72.45%–83.9%. The precision for intra-day ranged from 4.04% to 10.36% and for inter-day ranged from 8.69% to 9.8%. The trueness for intra-day and inter-day was in the range of −1.33%–9.38% and 0.58%–6.42%, respectively. The stability of paclitaxel was investigated under various conditions as followed: kept in an autosampler for 12 h, at −80°C for three months, and through three freeze-thaw cycles. The mean concentrations were all in the range of −7.55%–9.29% of nominal concentrations. This method could be applied to determine the concentrations of paclitaxel.

### Model Development and Evaluation

Population PK was performed using a non-linear mixed effect modeling technique (NONMEM, version7.3, ICON Development Solutions) (15). Two- and three-compartment models were evaluated to describe the concentration-time data and visual inspection of concentration-time plots. The candidate model was tested with various modifications. The model involved two-cycle data. Inter-individual variability assumed lognormal distributions which were modeled for each structural parameter as follows:

$$P_{i}=\text{Exp}\,\left(P_{T\,V}+\eta_{i}\right)$$

Where *P*_*i*_ represents the parameter for the *i*th individual, *P*_*TV*_ is the natural logarithm of the typical value of the parameter in the population, and η*_*i*_* are random inter-individual variables with mean zero and variance of ω^2^.

Residual variability was modeled as follows:

$$C_{i\,j}={\hat{C}}_{i\,j}\times \left(1+\varepsilon_{p\,r\,o\,p,i\,j}\right)$$

where C*_*ij*_* and ${\hat{C}}_{i\,j}$ represent the *j*th observed and predicted concentration, respectively, for the *i*th individual; ε*_*prop,ij*_* is the proportional portions of intra-individual variability with means of zero and variances of σ*_*prop*_^2^*.

The clinical relative covariates were examined for their impact on model parameters. Potential covariates included sex, age, weight, total bilirubin, albumin, serum creatinine, creatinine clearance, and co-administration with aidi injection. Covariate analysis included a two-step process. Firstly, the scatter plots of selected parameter estimates from the base model vs potential covariates were conducted. Secondly, a strategy including a stepwise forward addition and a backward deletion was applied to build up the full covariate model.

The model was validated by a bootstrap re-sampling technique. Monte Carlo method was used for random sampling with replacement of the original dataset for estimating variance and comparing it with the original population parameters. Visual predictive check (VPC) was applied for graphically assessing the appropriateness of the compartment model prediction. The 1,000 sets of concentration values were simulated and compared with the observed data to evaluate the predictive performance of the model.

### Exposure–Safety Analysis

The exposure–safety analysis was performed using a generalized linear regression modeling approach. Due to the insufficient data across different grades, the analyses were not stratified by grade. Safety data about neutropenia were collected from electronic medical records, which included any grade according to the NCI Common Terminology Criteria for Adverse Events (CTCAE) 4.0. The individual *post hoc* PK parameters and dosage were used to calculate the AUC. Generalized linear regression models were tested as appropriate to describe adverse effects.

### Statistical Analysis

Pharmacokinetics analysis was performed using NONMEM v7.3. The Stepwise Covariate Model (SCM) module in Perl-speaks-NONMEM (PSN) software (version 3.2.12) was used for covariate screening, identification, and model assessment. R 3.5.1 was applied to show graphs. All statistical tests were performed at a significant level of 0.05.

## Results

### Patients

A total of 349 data points from 45 subjects with squamous cell NSCLC in two cycles were analyzed for PK measurement. All the concentrations were above the limit of detection. Two patients dropped out of the trial in cycle 2 because they gave up the treatment. The baseline demographics (Table 1) showed that 91% of subjects were male. The median age of these individuals was 59 years, and their median creatinine clearance was 101.1 mL min^–1^.

**TABLE 1:** Patient characteristics at baseline.

Characteristics	Median (minimum–maximum)
Continuous variable	
No. of patients	45
Age (year)	59 (36–75)
Weight (kg)	71.5 (45–100)
Creatinine clearance (mL min^–1^)	101.1 (53.4–159.1)
Total bilirubin (μmol L^–1^)	8.4 (2.3–35.5)
Albumin (g L^–1^)	42.6 (30.7–50.5)
Serum creatinine (μmol L^–1^)	69 (30.1–152)

Classified variable	N
Combination with aidi injection (no/yes)	No: 45 in Cycle1/Yes:43 in Cycle2
Male, n%	41, 91%
Dose (210 mg/240 mg/270 mg/300 mg)	4/29/31/24

### PK Parameter Estimates

#### Model

The PK profile of paclitaxel liposome was adequately described by a three-compartment disposition model with first-order elimination. The model included parameters for central clearance (CL), the central volume (Vc), deep peripheral volume (V_*p*1_), shallow peripheral volume (V_*p*2_), and distribution clearance (Q). The final PopPK model included the following parameter relations:

CLi=EXP⁢(θ⁢CL+η⁢C⁢L)

The estimated population CL value of paclitaxel from the final PopPK model was 21.55 L h^–1^ (95% CI, 18.59–25). The estimated deep peripheral volume (V_*p*1_) was 44.15 (95% CI, 36.5–53.39). The Inter-individual variability was 20.65% for CL and intra-individual variability (σ_1_) expressed as the residual error was 44.55%. Median values of PopPK parameter estimates from bootstrapping were similar to the parameter estimates of the developed model with 95% CI. The final model sufficiently described the data. The final estimated parameters from the PopPK model were listed in Table 2.

**TABLE 2:** The final population pharmacokinetic parameters of paclitaxel liposome.

	Parameter	Population estimate	Bootstrap final model
Parameters	description	(95%Cl)	median (5, 95% percentiles)
Vc	Central volume (L)	0.9248 (0.7733–1.106)	0.8904 (0.7749–1.01)
V_*p*1_	Deep peripheral volume (L)	44.15 (36.5–53.39)	42.79 (35.99–50.36)
V_*p*2_	Shallow peripheral volume (L)	5.577 (4.838–6.429)	5.483 (4.043–6.839)
CL	Central clearance, CL (L h^–1^)	21.55 (18.59–25)	20.8 (18.56–22.86)
Q1	Distribution clearance between central and deep peripheral volume, Q1 (L h^–1^)	4.62 (3.915–5.453)	4.481 (3.803–5.175)
Q2	Distribution clearance between central and shallow peripheral volume, Q2 (L h^–1^)	15.85 (10.95–22.96)	15.38 (9.506–27.76)
Inter-individual variable (%) CL	Inter-individual variability of CL	20.65 (13.94–25.66)	19.99 (14.03–25.94)
Residual error(%) σ_1_	proportion residual error (%)	44.55 (40.62–48.17)	44.28 (40.56–48.1)

#### Model Validation

**FIGURE 2:**
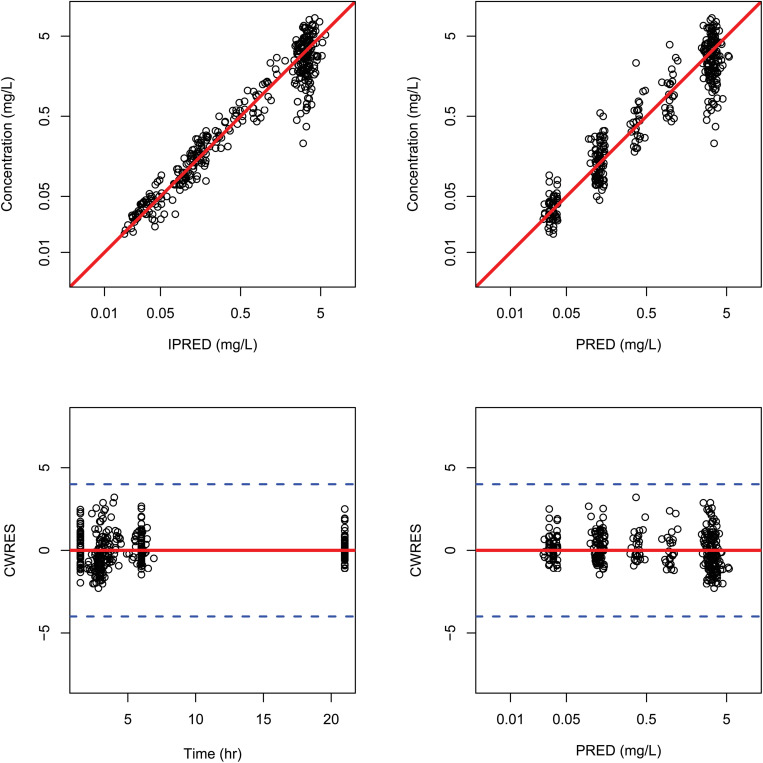
Goodness-of-fit plots for the final population pharmacokinetic model. Individual predicted plasma concentrations (IPRED) **(upper left panel)** and population predicted plasma concentrations (PRED) **(upper right panel)**; conditional weighted residuals versus times **(lower left panel)** and population residuals versus population predictions of total plasma paclitaxel **(lower right panel)**.

**FIGURE 3:**
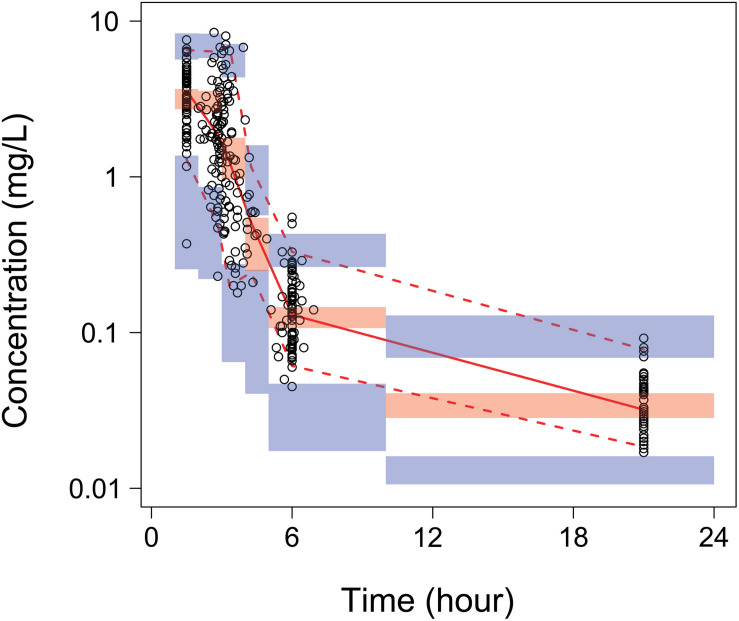
Visual predictive checks (VPCs) of total paclitaxel plasma concentrations for the final model. Open circles represent the individual observed concentrations. The solid line and dashed lines represent median and 95% confidence intervals of observed concentrations, separately. The shaded area is 95% confidence prediction intervals for the median (red) and the 2.5th and 97.5th percentiles of the results of 1,000 times simulation of the pharmacokinetic final model (blue).

The general goodness-of-fit plots were presented in Figure 2. There was a good agreement between predicted and observed concentrations of total plasma paclitaxel. No apparent bias was found in the residual plot. The VPCs for overall data also showed that the final model sufficiently described the data (Figure 3).

#### Covariate Analysis

**FIGURE 4:**
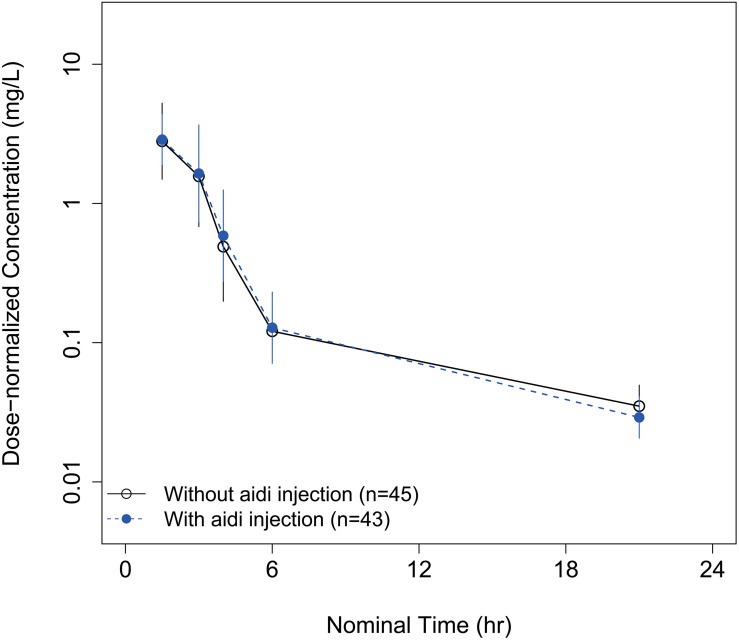
The mean total plasma concentration–time curves of paclitaxel without concomitant use of aidi injection in cycle 1 (solid line) and with concomitant use of aidi injection in cycle 2 (dashed line).

Parameter–covariate relationships were investigated. Age, sex, body weight, total bilirubin, albumin, serum creatinine, and creatinine clearance had no statistically significant influence on the inter-individual variable of CL. In the cycle 2 regimen, aidi injection was administrated before the infusion of Lipusu. There was no significant PK change of total plasma paclitaxel observed between the two cycles (Figure 4). No covariate effects were found on any of the model parameters.

#### Exposure–Safety Analysis

**FIGURE 5:**
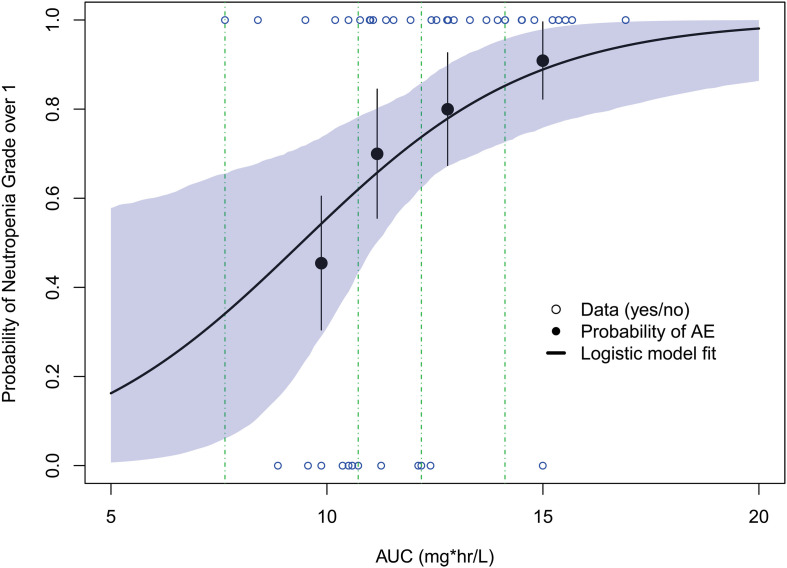
Model-predicted exposure–safety relationships for the probability of neutropenia grade over 1. Open circles reflect the observed events; filled circles are the observed responder probability; the error bars are the standard error for quantiles (at 10 percentiles) of exposures (plotted at the median value within each quantile). The shaded area represents the 90% confidence interval from bootstrapping.

The probability of a patient developing a neutropenia grade over 1 increased with the exposure to paclitaxel (AUC) (Figure 5). A generalized linear regression model adequately described the data. The final model was applied:

logit[P(NE>1)]=a+b×AUC

where logit is the logit transform; P(NE > 1) represents the probability of a patient with neutropenia grade over 1.

The parameter estimates from the final logistic model with the probability of neutropenia were presented in Table 3. The results suggested higher AUC was significantly associated with a higher probability of a neutropenia grade over 1 (*p* = 0.0469).

**TABLE 3:** Model parameters for the probability of adverse effects.

Adverse			Standard	
effect	Parameter	Estimated	error	*P* (>|z|)
Neutropenia grade > 1	a	-3.5008	2.1942	0.1106
	b	0.372	0.1872	0.0469

## Discussion

This is the first report on a PopPK model of paclitaxel liposome (Lipusu), which well described the PK profile of Lipusu in patients with squamous cell NSCLC. In our study, the PopPK of Lipusu showed a three-compartment model profile with first-order elimination from the central compartment. The total paclitaxel concentrations in plasma were measured as the dependent variable, including paclitaxel-liposome complex and free and protein-bound paclitaxel. The total paclitaxel concentrations showed a fast decline in the first phase followed by a relatively slow decline in the second phase, which may suggest a rapid distribution and a slow clearance from the peripheral compartment. The estimated population median CL is 21.55/1.84 (median body surface area)(11.71) L h^–1^ m^–2^, comparable with the CL of 12.3 ± 2.7 L h^–1^ m^–2^ reported in a publicly available report about the PK of Lipusu in patients with various tumors (16).

Various intrinsic factors, including age, sex, body weight, total bilirubin, albumin, serum creatinine, and creatinine clearance, and extrinsic factor such as concomitant aidi injection use had been evaluated, however, no significant parameter-covariate relationships were found. Paclitaxel is mainly metabolized by the liver and eliminated through biliary excretion (17). The hepatic function had an influence on conventional paclitaxel exposure (18). However, the present study only included patients with normal hepatic function, except for one patient with slightly elevated total bilirubin (35.5 μmol L^–1^). In addition, squamous cell lung cancer predominated in men, so more than 90% of the subjects in our study were male. This may partially explain the negative covariate effect.

Aidi injection is a traditional Chinese medicine, which is composed of cantharidin, ginsenoside, astragaloside, and acanthopanacis senticosi polysaccharide (19). It was widely used in China as an adjuvant treatment due to its anti-angiogenesis and immunoregulation effects. Aidi injection had been observed to have an inhibitory effect on the activity of CYP450 2C8 and 3A4 enzyme in the human liver microsome (20). However, no information is available on the effects of aidi injection on the PK of paclitaxel *in vivo*. Our data did not show the differences between PK profile of paclitaxel with and without aidi injection. Not only that, patients with a short hospitalization period for chemotherapy had no chance to receive multiple doses of aidi injection. Our results could provide a reference for co-administration with the two agents in clinical practices.

The final popPK model for paclitaxel liposome was evaluated using multiple methods. Goodness-of-fit plots showed a good agreement between observed and predicted data. Internal model evaluations VPC and bootstrap assessments gave evidence that the model was stable and adequate to describe the observed data. This popPK model was appropriate for subject exposure estimates for use in the exploratory evaluation of relationships between exposure and safety. Our model described a generalized significantly linear relationship between the exposure to paclitaxel and neutropenia grade over 1. This model suggested the profile of drug concentration-time vs the proliferation of the sensitive cell, which was common to chemotherapy drugs (21). The preliminary results revealed that the frequency of neutropenia may be dose- or schedule-dependent. At present, the maximum tolerated dose of Lipusu has not been reported. However, a Phase IV study for dose escalation with the designed maximum dose up to 300 mg m^–2^ has been carried out (22). This exploration of the relationship exemplified its role in dose recommendation for future studies.

There are some limitations to our study. First, it was a single-center study. Limited patients with normal hepatic and renal functions were included. Second, our popPK model requires external validation. Its predictive power needs to be further explored. Regardless, it is the first description of liposomal paclitaxel PK in NSCLC patients, which could provide a good foundation for future investigations.

## Conclusion

The PopPK of paclitaxel liposome were well described by a three-compartment model with first-order elimination. No significant PK changes of paclitaxel were observed in the presence of the traditional Chinese medicine, aidi injection. The exposure–safety analysis showed a higher probability of neutropenia was associated with higher exposure to paclitaxel.

## Data Availability Statement

The datasets generated for this study are included in the article/Supplementary Material or in the FigShare doi: 10.6084/m9.figshare.13554704.v1.

## Ethics Statement

The studies involving human participants were reviewed and approved by the Institutional Review Board of Cancer Hospital, Chinese Academy of Medical Sciences. The patients/participants provided their written informed consent to participate in this study.

## Author Contributions

GL, HZ, WC, JuY, XH, and YW designed the experiments. HZ, JiY, JuY, ML, YZ, XS, YM, XH, and YW collected the data. HZ, WC, and GL analyzed the data. HZ and WC wrote the manuscript. KD was responsible for bioanalytical results. All authors reviewed and approved the final manuscript.

## Conflict of Interest

KD was employed by the company Iphase Pharma Services. The remaining authors declare that the research was conducted in the absence of any commercial or financial relationships that could be construed as a potential conflict of interest.

## References

[1] Cristiano MC Froiio F Spaccapelo R Mancuso A Nisticò SP Udongo BP Sulforaphane-loaded ultradeformable vesicles as a potential natural nanomedicine for the treatment of skin cancer diseases. *Pharmaceutics* (2019) 12:6. 10.3390/pharmaceutics12010006 31861672 PMC7023209

[2] Di Francesco M Primavera R Fiorito S Cristiano MC Taddeo VA Epifano F acronychiabaueri analogue derivative-loaded ultradeformable vesicles: physicochemical characterization and potential applications. *Planta Med.* (2017) 83:482–491. 10.1055/s-0042-112225 27542175

[3] Cilurzo F Cristiano MC Di Marzio L Cosco D Carafa M Ventura CA. Influence of the supramolecular micro-assembly of multiple emulsions on their biopharmaceutical features and in vivo therapeutic response. *Curr. Drug Targets* (2015) 16:1612–1622. 10.2174/138945011614151119124234 26601721

[4] Ye L He J Hu Z Dong Q Wang H Fu F Antitumor effect and toxicity of Lipusu in rat ovarian cancer xenografts. *Food Chem Toxicol.* (2013) 52:200–6. 10.1016/j.fct.2012.11.004 23149094

[5] Szebeni J Muggia FM Alving CR. Complement activation by Cremophor EL as a possible contributor to hypersensitivity to paclitaxel: an in vitro study. *J Natl Cancer Inst.* (1998) 90:300–6. 10.1093/jnci/90.4.300 9486816

[6] Chen Q Zhang QZ Liu J Li LQ Zhao WH Wang YJ Multi-center prospective randomized trial on paclitaxel liposome and traditional taxol in the treatment of breast cancer and non-small-cell lung cancer. *Zhonghua Zhong Liu Za Zhi.* (2003) 25:190–2. 12795852

[7] Xu X Wang L Xu HQ Huang XE Qian YD Xiang J. Clinical comparison between paclitaxel Liposome (Lipusu^®^) and paclitaxel for treatment of patients with metastatic gastric cancer. *Asian Pac J Cancer Prev.* (2013) 14:2591–4. 10.7314/apjcp.2013.14.4.2591 23725180

[8] Database of Abstracts of Reviews of Effects (DARE): Quality-assessed Reviews [Internet]. Effectiveness and safety of paclitaxel liposomes and carboplatin for ovarian cancer: a systematic review. 2012. New York (UK): Centre for Reviews and Dissemination (UK); 1995 Available from: https://www.ncbi.nlm.nih.gov/books/NBK121506/

[9] Hu L Liang G Yuliang W Bingjing Z Xiangdong Z Rufu X. Assessing the effectiveness and safety of liposomal paclitaxel in combination with cisplatin as first-line chemotherapy for patients with advanced NSCLC with regional lymph-node metastasis: study protocol for a randomized controlled trial (PLC-GC trial). *Trials.* (2013) 14:45. 10.1186/1745-6215-14-45 23413951 PMC3599280

[10] Zhang Q Huang XE Gao LL. A clinical study on the premedication of paclitaxel liposome in the treatment of solid tumors. *Biomed Pharmacother.* (2009) 63:603–7. 10.1016/j.biopha.2008.10.001 19019625

[11] Wang X Zhou J Wang Y Zhu Z Lu Y Wei Y A phase I clinical and pharmacokinetic study of paclitaxel liposome infused in non-small cell lung cancer patients with malignant pleural effusions. *Eur J Cancer.* (2010) 46:1474–80. 10.1016/j.ejca.2010.02.002 20207133

[12] Xiao Z Wang C Zhou M Hu S Jiang Y Huang X Clinical efficacy and safety of Aidi injection plus paclitaxel-based chemotherapy for advanced non-small cell lung cancer: a meta-analysis of 31 randomized controlled trials following the PRISMA guidelines. *J Ethnopharmacol.* (2019) 228:110–22. 10.1016/j.jep.2018.09.024 30243827

[13] Xie G Cui Z Peng K Zhou X Xia Q Xu D. Aidi injection, a traditional Chinese medicine injection, could be used as an adjuvant drug to improve quality of life of cancer patients receiving chemotherapy: a propensity score matching analysis. *Integr Cancer Ther.* (2019) 18:1534735418810799. 10.1177/1534735418810799 30482065 PMC6432675

[14] Sottani C Minoia C D’Incalci M Paganini M Zucchetti M. High-performance liquid chromatography tandem mass spectrometry procedure with automated solid phase extraction sample preparation for the quantitative determination of paclitaxel (Taxol) in human plasma. *Rapid Commun. Mass Spectrom.* (1998) 12:251–5. 10.1002/(SICI)1097-0231(19980314)12:5<251::AID-RCM145>3.0.CO;2-Z 9519477

[15] European Medicines Agency. *Guideline on Reporting the Results of Population Pharmacokinetic Analyses.* Amsterdam: European Medicines Agency (2007).

[16] Qian J Wang YX Yu YQ Li J. A comparison of pharmacokinetics between paclitaxel liposome for injection and commercial paclitaxel injection in patients with cancer. *Tumor.* (2011) 31:1103–7. 10.3781/j.issn.1000-7431.2011.12.010

[17] Dw VA Beijnen JH Schellens JH. Cellular and clinical pharmacology of the taxanes docetaxel and paclitaxel - a review. *Anticancer Drugs.* (2014) 25:488–94. 10.1097/CAD.0000000000000093 24637579

[18] Panday VR Huizing MT Willemse PH De Graeff A ten Bokkel Huinink WW Vermorken JB Hepatic metabolism of paclitaxel and its impact in patients with altered hepatic function. *Semin Oncol.* (1997) 24:S11–34. 9314297

[19] Zhang MM Liu YL Chen Z Li XR Xu QM Yang SL. A new triterpenoid saponin from aidi injection. *Chinese Herb Med.* (2012) 4:84–6. 10.3969/j.issn.1674-6384.2012.02.002

[20] Liu LY Han YL Yu Q Zhu JH Guo C. In vitro inhibition of 4 anti-tumor traditional Chinese medicine injections on activities of 7 main cytochrome P450s in human liver microsome. *Chin J Clin Pharmacol Ther.* (2014) 19:522–7.

[21] Friberg LE Henningsson A Maas H Nguyen L Karlsson MO. Model of chemotherapy-induced myelosuppression with parameter consistency across drugs. *J Clin Oncol.* (2002) 20:4713–21. 10.1200/JCO.2002.02.140 12488418

[22] ClinicalTrials.gov. *Dose Escalation and Pharmacokinetic Study of Paclitaxel Liposome Injection in Treating Patients With Advanced Solid Tumor After Failure From Conventional Treatments.* (2014). Available online at: https://clinicaltrials.gov/ct2/show/NCT01994031 (accessed October 7, 2016).

