# Supplementary material for: Population Pharmacokinetics and Exposure–Safety Relationship of Paclitaxel Liposome in Patients With Non-small Cell Lung Cancer
Source: Front Oncol. 2021 Feb 5;10:1731. doi: 10.3389/fonc.2020.01731 (PMC7892953; doi:10.3389/fonc.2020.01731)
Supplement: Supplementary file 1 [file Data_Sheet_1.docx]

;; 1. Based on: run11e

;; 2. Description: 3 compartments PK model

;; x1. Author: user

;; 3. Label:

$PROBLEM PK

$INPUT ID PID TIME DV AMT RATE ADAD DOSE AGE WT TB SCR CLCR GEND ALB

$DATA run4.csv IGNORE=#

$SUBROUTINE ADVAN6 TOL=3

$MODEL

COMP(CENTRAL,DEFDOSE)

COMP (PERIPH1)

COMP (PERIPH2)

$PK

V1=EXP(THETA(1)+ETA(1)) ; volume 1

V2=EXP(THETA(2)+ETA(2)) ; volume 2

V3=EXP(THETA(3)+ETA(3)) ; volume 3

CL1=EXP(THETA(4)+ETA(4)) ; clearance 1

CL2=EXP(THETA(5)+ETA(5)) ; clearance 2

CL3=EXP(THETA(6)+ETA(6)) ; clearance 2

K10=CL1/V1

K12=CL2/V1

K13=CL3/V1

K21=CL2/V2

K31=CL3/V3

$DES

DADT(1) = A(2)*K21+A(3)*K31-A(1)*(K10+K12+K13)

DADT(2) = A(1)*K12-A(2)*K21

DADT(3) = A(1)*K13-A(3)*K31

$ERROR

IPRED=F

IRES=DV-IPRED

DEL=0

If (dv .EQ. 0) DEL=1

IWRES=(1-DEL)*IRES/(DV+DEL)

Y=F*(1+EPS(1))

$THETA

(-10, -0.0611,10) ; V1

(-10, 3.8,10) ; V2

(-10, 1.74,10) ; V3

(2, 3.09,4) ; CL1

(-10, 1.55,10) ; CL2

(-10, 2.78,10) ; CL3

$OMEGA

0 FIX ; IIV V1

0 FIX ; IIV V2

0 FIX ; IIV V3

0.0427 ; IIV CL1

0 FIX ; IIV CL2

0 FIX ; IIV CL3

$SIGMA

0.199 ; Proportional error PK

$ESTIMATION METHOD=1 INT MAXEVAL=9999 NOABORT SIG=3 SIGL=9 PRINT=1 POSTHOC

$COVARIANCE

$TABLE ID TIME RATE ADAD GEND AMT DOSE AGE WT TB SCR CLCR ALB PRED CWRES RES IPRED IWRES PRED CWRES RES IPRED IWRES DV CL1 CL2 CL3 V1 V2 V3 ETA1 ETA2 ETA3 ETA4 ETA5 ETA6 NOPRINT ONEHEADER FILE=RES11g
